# Supplementary material for: Assessing elevated pressure impact on photoelectrochemical water splitting via multiphysics modeling
Source: Nat Commun. 2024 Jun 10;15:4944. doi: 10.1038/s41467-024-49273-2 (PMC11164907; doi:10.1038/s41467-024-49273-2)
Supplement: Supplementary file 1 — Supplementary Information [file 41467_2024_49273_MOESM1_ESM.pdf]

Supplementary information

Assessing elevated pressure impact on photoelectrochemical water splitting via multiphysics modeling

Feng Liang,[1] Roel van de Krol,[1,2] and Fatwa F. Abdi[1,3*]

[1] Institute for Solar Fuels, Helmholtz-Zentrum Berlin für Materialien und Energie GmbH, Hahn-Meitner-Platz 1, 14109, Berlin, Germany

[2] Technische Universität Berlin, Department of Chemistry, Straße des 17. Juni 124, 10623, Berlin, Germany

[3] School of Energy and Environment, City University of Hong Kong, 83 Tat Chee Avenue, Kowloon, Hong Kong SAR, China

Correspondence and requests for materials should be addressed to Fatwa F. Abdi (email: ffabdi@cityu.edu.hk)

Table S1. The coefficients for the generic fitting equation (equation 1) of D_{O_2}, D_{H_2}, N_{O_2} and N_{H_2}.

	D_{O_2}	D_{H_2}	N_{O_2}	N_{H_2}
a	21.13	10.25	1436.72	-10037.8
b_1	-0.68	2.15	0.00002	0
b_2	2	-3.6	12	0
b_3	0	0	-0.0014	0.14
b_4	0	0	3	4
b_5	0	-0.000075	0	0
b_6	0	-11	0	0
c_1	-26.63	-12.24	0	439.24
c_2	1.55	0.11	0	0.01
c_3	0	0	-32.61	0
c_4	0	0	0.2	0
d_1	69.93	18.68	-1387.89	10069.4
d_2	-1.1	-1.2	-0.08	0.01
d_3	0	-217.06	434.28	0
d_4	0	-4	-3	0

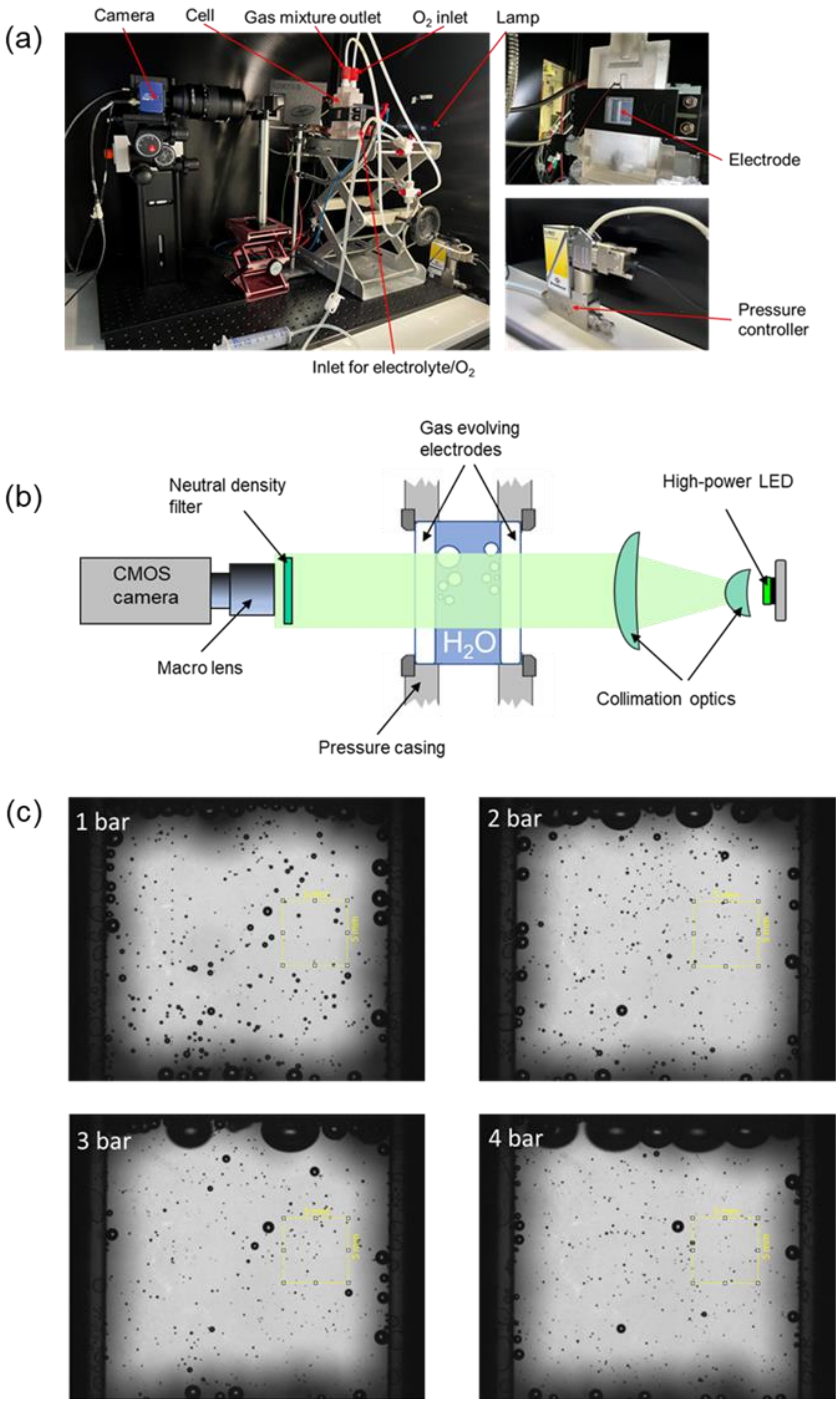

Figure S1. (a) Photographs and (b) schematic illustration of the experimental setup for bubble observation. (c) O_2 bubble visualization results for the operating pressure of 1-4 bar. The region-of-interest (ROI) for each condition are marked in yellow.

Supplementary Note 1 – Determining bubble formation efficiency $\boldsymbol{\eta_{\mathrm{bub}}}$ with $\boldsymbol{D_{\mathrm{bub}}}$ and $\boldsymbol{N_{\mathrm{bub}}}$

The bubble formation efficiency, η_{bub}, was determined using the values of bubble diameter (D_{bub}) and the number density of bubble (N_{bub}). Here, we provide the step-by-step procedure of the calculation by taking the O_2 bubble formation efficiency, η_{bub,O_2}, as an example.

First, the number of moles in an O_2 bubble ($n_{\mathrm{bub,O_2}}$) can be estimated with the ideal gas equation by taking into account the pressure difference due to surface tension between the spherical bubble surface (i.e., the Laplace pressure) and the vapor pressure of water.[1]

$$n_{\mathrm{bub,O_2}} = \frac{(P_0 - P_{\mathrm{H_2O}} + 4\gamma/D_{\mathrm{bub,O_2}})V_{\mathrm{bub,O_2}}}{RT} \tag{S1}$$

Here, $V_{\mathrm{bub,O_2}} = \frac{4}{3}\pi\left(\frac{D_{\mathrm{bub,O_2}}}{2}\right)^3$ is the volume of a gas bubble, P_0 is the operational pressure in the PEC cell, $P_{\mathrm{H_2O}} = \frac{3344}{101325}P_0$ is the vapor pressure of water in the bubble, $\gamma = 0.072$ Pa m is the bubble interface surface tension, R is the molar gas constant, and $T = 298.15$ K is the ambient temperature.

The total observed molar gas flow rate ($\dot{\mathbf{n}}_{\mathrm{O_2}}$) at the region of interest (i.e., the center of the sample) can be calculated based the following equation:

$$\dot{\mathbf{n}}_{\mathrm{O_2}} = \frac{\langle \mathbf{u}_{\mathrm{bub}}\rangle \langle N_{\mathrm{bub,O_2}}\rangle \langle n_{\mathrm{bub,O_2}}\rangle}{h} \tag{S2}$$

where h is the height of each photographic frame (this value is 40 mm in Sillen's experiment,[2] see section 3.2.2 of the study) and $\mathbf{u}_{\mathrm{bub}}$ is the rising velocity of bubbles; we used the Stokes terminal velocity of O_2 bubbles for simplicity, as good agreement between the Stokes terminal velocity of O_2 bubble and the measured data has been shown by Holmes-Gentle et al.[3]

$$\mathbf{u}_{\mathrm{bub}} = \frac{2\mathbf{g}(\rho_{\mathrm{g}} - \rho_{\mathrm{l}})}{9\mu_{\mathrm{L}}}\left(\frac{D_{\mathrm{bub,O_2}}}{2}\right)^2 \tag{S3}$$

ρ_g and ρ_l are the density for O_2 and electrolyte, respectively, $\mathbf{g}$ is the acceleration of gravity (9.81 m^2/s), and μ_L is the dynamic viscosity of the electrolyte.

$\dot{\mathbf{n}}_{O_2}$ can then be compared with the theoretical molar production rate of O_2 ($\dot{\mathbf{n}}_{O_2,\mathrm{theory}}$) assuming uniform current density distribution at the anode and 100% faradaic efficiency.

$$\dot{\mathbf{n}}_{O_2,\mathrm{theory}} = \frac{\mathbf{j}_{\mathrm{loc}} A_{\mathrm{el}}}{n_e F} \tag{S4}$$

where $\mathbf{j}_{\mathrm{loc}}$ denotes the local current density, A_{el} is the surface area of the electrode, n_e and F are the electron stoichiometry in the reaction and the Faraday constant, respectively.

Finally, the O_2 bubble formation efficiency can be calculated by taking the ratio of $\dot{\mathbf{n}}_{O_2}$ and $\dot{\mathbf{n}}_{O_2,\mathrm{theory}}$.

$$\eta_{\mathrm{bub},O_2} = \frac{\dot{\mathbf{n}}_{O_2}}{\dot{\mathbf{n}}_{O_2,\mathrm{theory}}} \tag{S5}$$

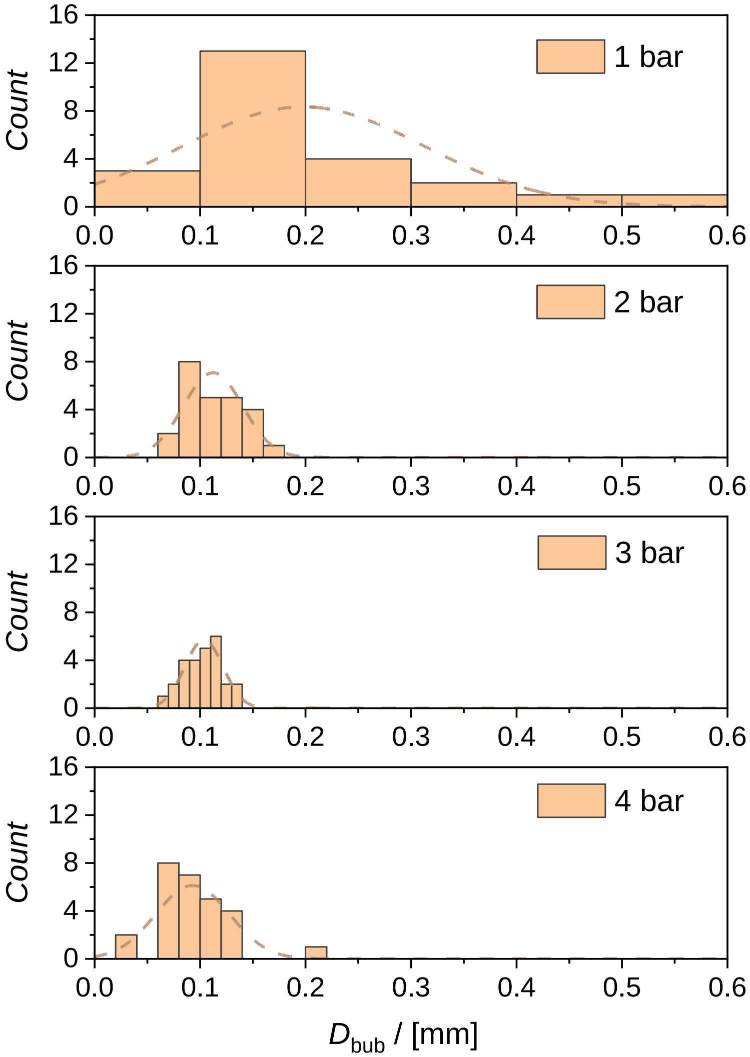

Figure S2. Histograms of measured oxygen bubble diameters (D_{bub}) at different operating pressures.

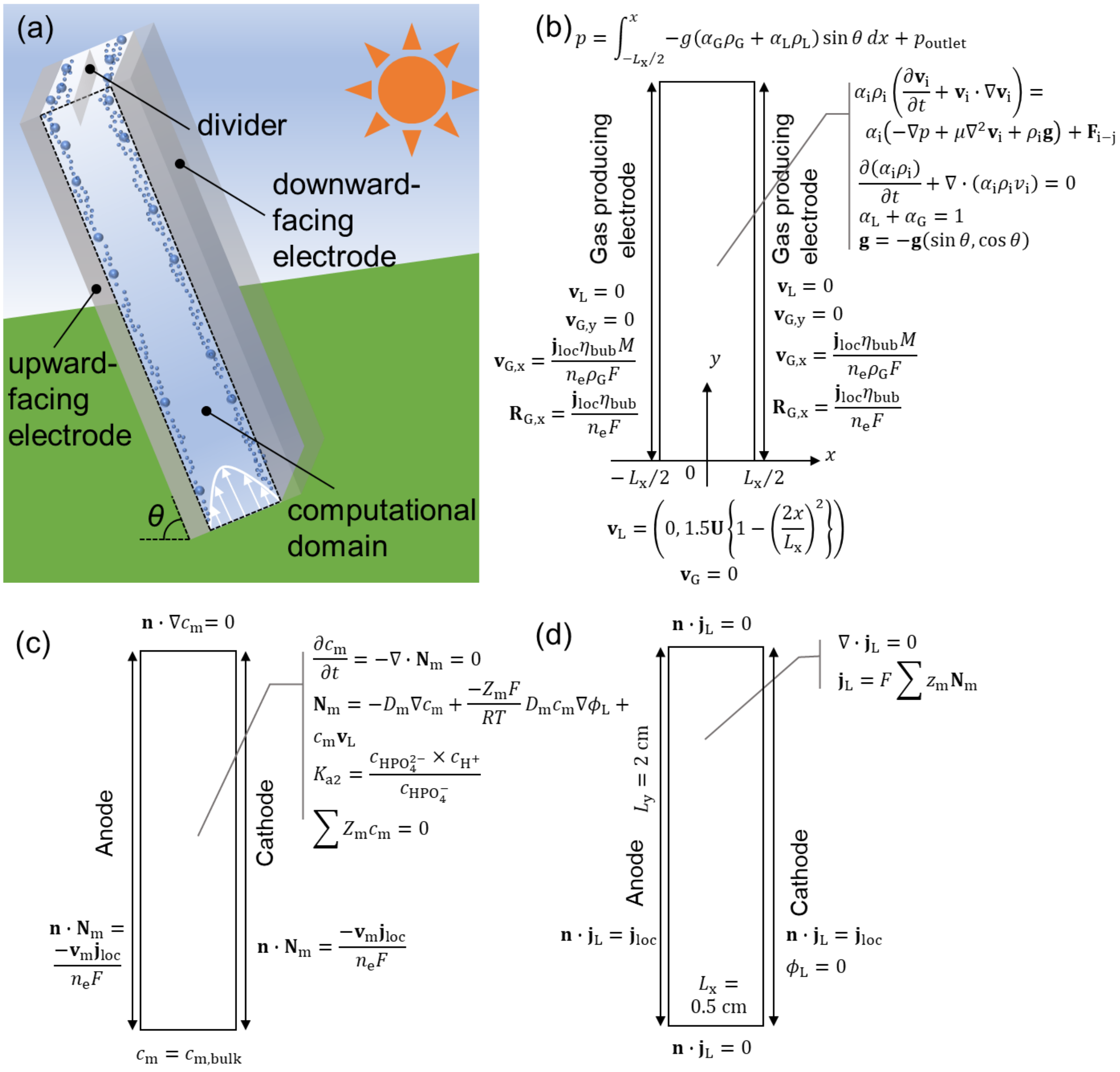

Figure S3. (a) Schematic illustration of a membrane-free PEC water-splitting device and the boundary conditions related to (b) two-phase fluid flow, (c) mass transport of diluted species, and (d) electrochemistry in our multiphysics model.

Supplementary Note 2 – Model validation

A flow cell was constructed and used for the model validation, as shown in the schematic and pictures in Fig. S4a and b. Two parallel electrodes (the same ones used for bubble observation, see Methods section) were placed vertically inside the flow cell as indicated. 1M KOH (pH 14) was used as the electrolyte and circulated using a gear pump (LAB-9, Garther Industrie). To ensure a well-distributed liquid flow profile between the two electrodes, the liquid flow was fed through a fluid distributor region before reaching the reactor (see Fig. S4a).

We first examined the flow velocity between the two parallel electrodes using particle image velocimetry (PIV, LaVision). The distance between the two electrodes in our experimental setup is 0.4 cm, which is slightly lower than in our model ($L_{\mathrm{x}} = 0.5$ cm, see Fig. S3d); we therefore adjusted L_{x} during our simulations to mimic the experimental conditions. The PIV results are shown in Fig. S4c. A parabolic flow profile was observed between the two electrodes, and the average flow velocity in the channel was determined to be 0.3 cm s^{-1}. Using this value as the boundary condition for the inlet velocity, we simulated the flow velocity as shown in Fig. S4d. Following that, we conducted multiphase flow simulations and bubble observation experiments. The camera setup and the parameters used for the bubble shadowgraphy here is the same as reported in Methods, but note that the electrodes were observed from the side during these experiments, which is different from Fig. S1c. A better view of the bubble footage can be found in Supplementary videos S1-S4.

The simulated bubble curtains (defined as the contour line of 99% volume fraction of the gas phase) from our multiphase flow model were then compared against those observed from our measurements (determined from the largest bubble diameter at selected locations of the electrode).

The comparisons are shown in Fig. S5a-d, indicating that the simulated results and the trend with pressure agree relatively well with the measured values. We briefly note that measurement at certain locations were not possible since bubbles located at the background blocked the view. This is especially the case at the top of the electrode, where electrical contacts were made. The contact was done by fixing a copper wire on the electrodes, which was then covered with silicon glue. Bubbles were likely to be accumulated in these regions during our experiments, as can be seen from the shadows at the top of Supplementary videos S1-S4.

We also validated our model by comparing the dissolved gas concentration to that obtained from the empirical relationship reported by Shibata and Vogt.[4,5] As shown in Fig. S6, both the dissolved O_2 and H_2 concentrations obtained from our model are much higher than the solubility values, and they are in relatively good agreement with the empirical supersaturated concentration; slight deviation can be explained by the fact that no bubble formation is considered in the empirical correlation.

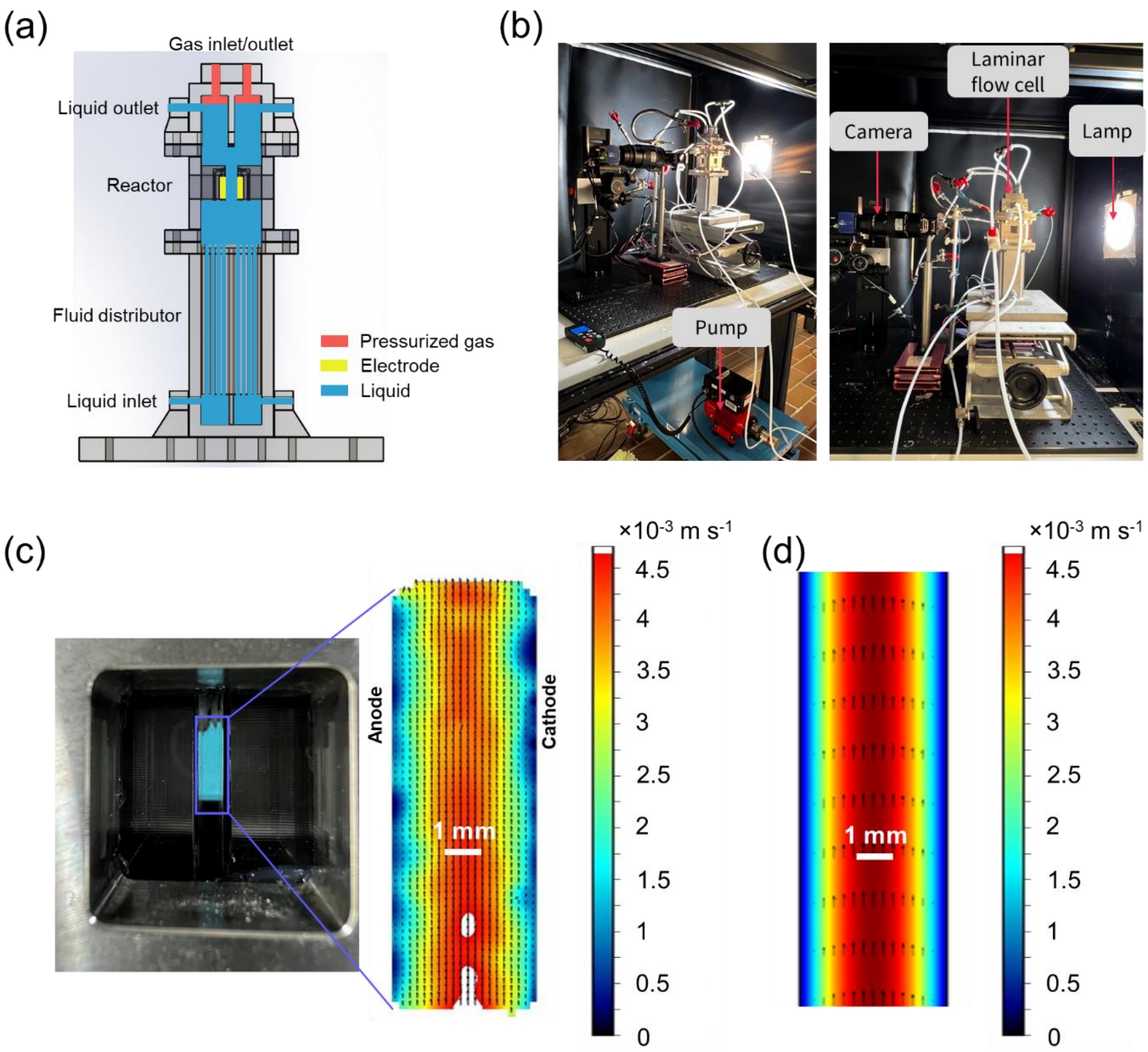

Figure S4. Experimental validation of the multiphase flow model. (a) Schematic illustration of the experimental setup. (b) Photographs of the experimental setup. (c) Photograph of the observation window (left), and the region marked in blue is the region-of-interest of flow examination. The colormap shown represents the measured liquid velocity profile from particle image velocimetry (PIV). (d) The simulated liquid velocity profile using our multiphysics model by taking the velocity value from PIV test as the boundary condition.

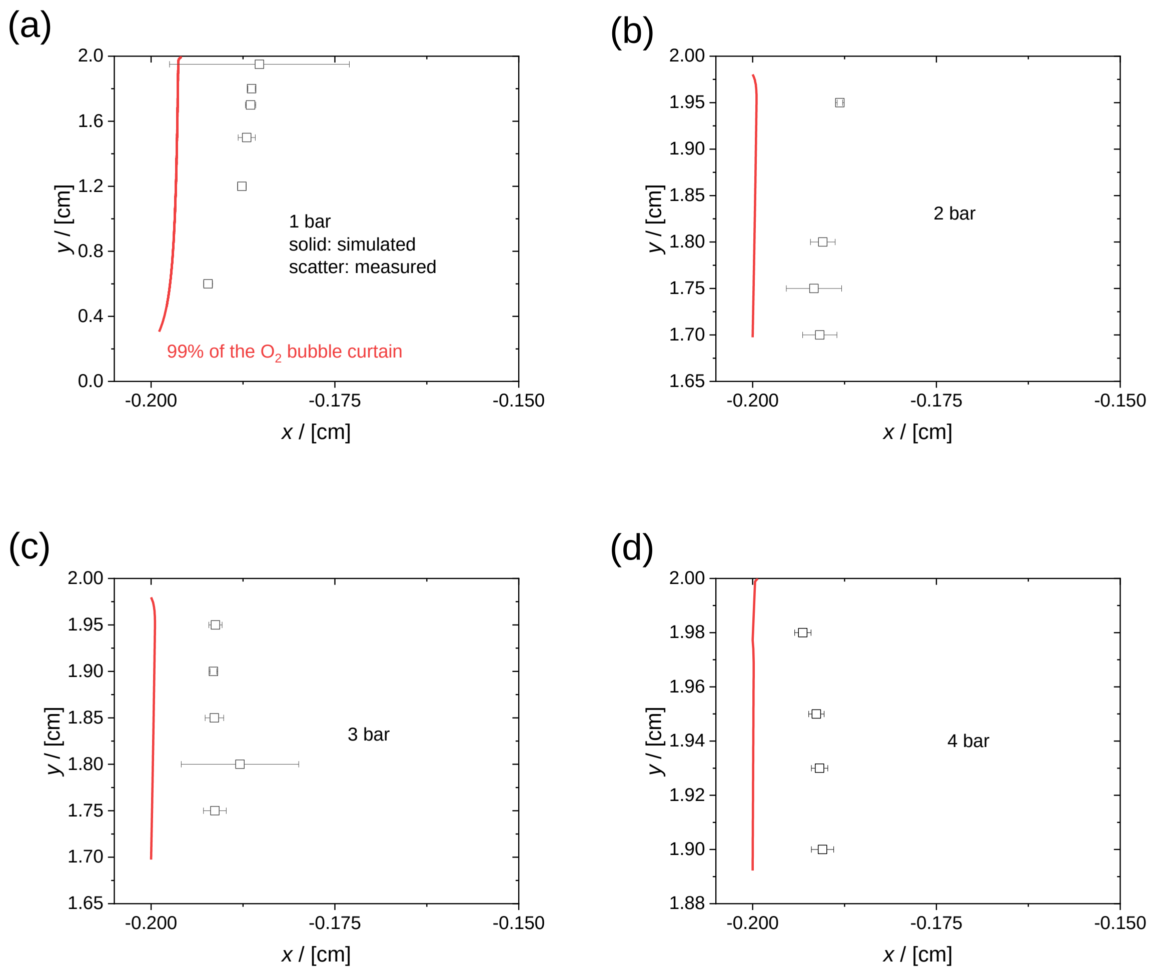

Figure S5. Comparisons of the simulated and measured oxygen bubble curtains. Results at various pressure are shown: (a) 1 bar, (b) 2 bar, (c) 3 bar, and (d) 4 bar. The simulated bubble curtain is defined as the 99% of the dispersed phase (ϕ_{d}). The distance between the two electrodes is 0.4 cm, $x = 0$ cm is the middle of the two electrodes, $x = -0.2$ cm is therefore the location of the anode. Error bars are the standard deviations of at least three measurements. The flow cell is pressurized using N_2 during the experiments.

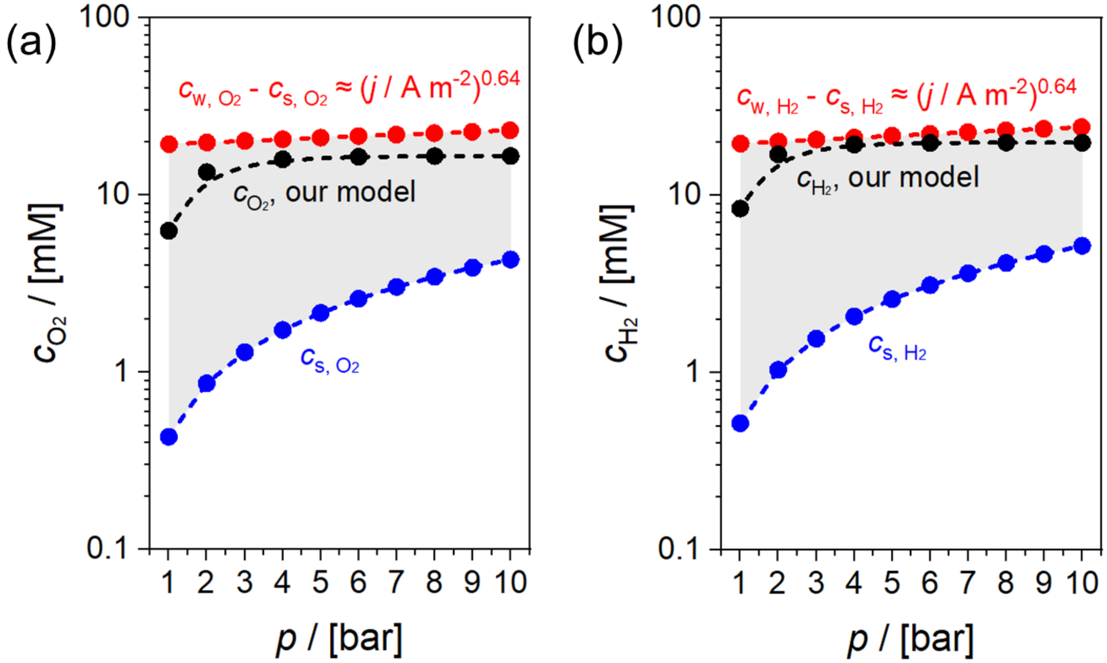

Figure S6. Supersaturated concentration of (a) O_2 and (b) H_2 gases in the vicinity of electrodes. c_w is the predicted concentration of O_2 or H_2 in the vicinity of electrode based on the empirical equation in literature.[4,5] c_s is the molar solubility of gas in water, which is calculated based on Henry's law: $c_s = p_g/K_H$, in which p_g is the partial pressure of the products. Henry's constants for oxygen and hydrogen are $K_{H,O2} = 769.2 \text{ atm M}^{-1}$ and $K_{H,H2} = 1282.05 \text{ atm M}^{-1}$, respectively, [6,7] assuming that the solution is pure water and the temperature is 298 K. $c_{O_2,\text{our model}}$ and $c_{H_2,\text{our model}}$ are the simulated O_2 and H_2 concentrations using our model, respectively. **j** is the current density, which is 10 mA cm^{-2} in this case.

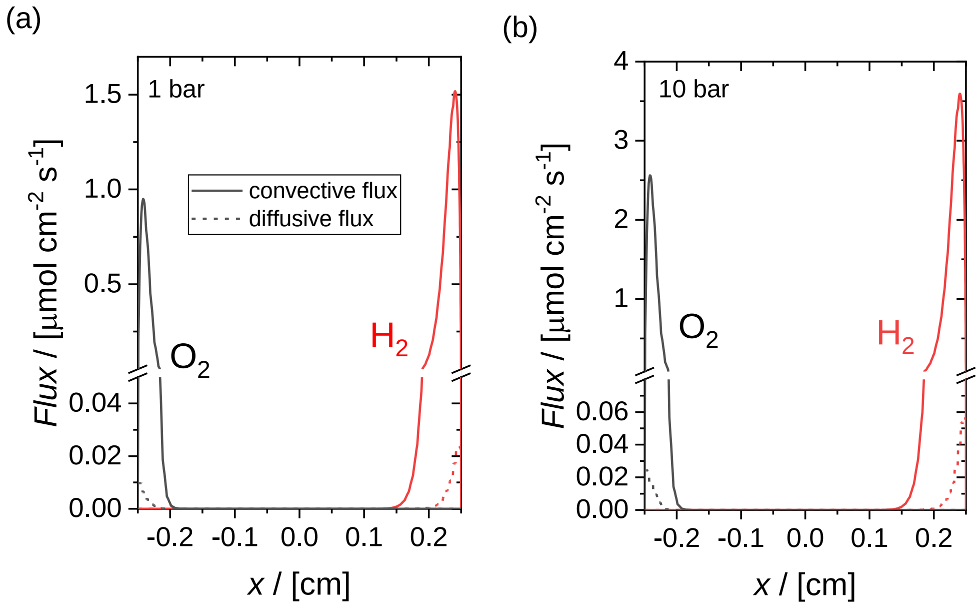

Figure S7. Molar flux of O_2 (black) and H_2 (red) at the device outlet from the convective (solid) and diffusive (dashed) contribution at the operating pressure of (a) 1 bar and (b) 10 bar.

Supplementary Note 3 – Step-by-step determination of the bubble-induced optical loss

To quantify the bubble-induced optical loss ($f_{\text{opt. loss}}$), we first determined the number of bubbles from the simulated gas volume fraction (ϕ_d) that was obtained from our multiphysics simulation. ϕ_d represents the ratio between the total volume of bubbles to the total volume. If we consider a single rectangular mesh element of $dx \times dy$ within our 2D computational domain, see illustration in Fig. S8, ϕ_d at each (*x*,*y*) coordinate in the domain can be written as:

$$\phi_\text{d}(x, y) = \frac{c_\text{bub}(x,y)A_\text{bub}}{dx\ dy} \tag{S6}$$

$c_\text{bub}(x, y)$ is the bubble count at a particular (x, y) coordinate, and A_bub is the projected area of a single bubble at a particular operating pressure, which can be calculated from the pressure-dependent D_bub (equation 1).

$$A_\text{bub} = \pi\left(\frac{D_\text{bub}}{2}\right)^2 \tag{S7}$$

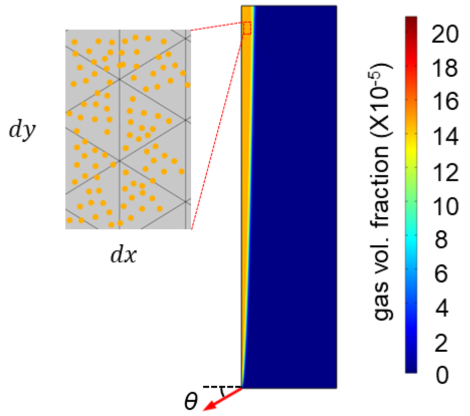

Figure S8. A closer look at the gas volume fraction (ϕ_d) in our multiphase simulations. To calculate the bubble count, we consider bubbles with a uniform diameter within each region of $dx \times dy$.

Re-arranging equations S6 and S7 results in:

$$c_{\mathrm{bub}}(x,y) = \frac{\phi_{\mathrm{d}}(x,y)\,dx\,dy}{\pi\left(\frac{D_{\mathrm{bub}}}{2}\right)^2} \tag{S8}$$

The total number of bubbles existed in the whole domain C_{bub} is:

$$C_{\mathrm{bub}} = \int_{-L_{\mathrm{y}}/2}^{L_{\mathrm{y}}/2}\int_{-L_{\mathrm{x}}/2}^{L_{\mathrm{x}}/2} \frac{\phi_{\mathrm{d}}(x,y)\,dx\,dy}{\pi\left(\frac{D_{\mathrm{bub}}}{2}\right)^2} \tag{S9}$$

We then calculated the fraction of light that is transmitted through the bubble plume ($f_{\mathrm{opt.\ trans.}}$) using a method modified from the literature.[3]

$$f_{\mathrm{opt.\ trans.}} = \exp\left(-\frac{K_{\mathrm{a}}al}{4}\right) \tag{S10}$$

where al is a dimensionless parameter comprising of the interfacial area per unit volume of the dispersion, a, and the path length of the light, l. This dimensionless parameter can be calculated using the following:

$$al = \frac{\langle C_{\mathrm{bub}}\rangle\langle D_{\mathrm{bub}}\rangle}{L_{\mathrm{y}}} \tag{S11}$$

K_{a} is the total scattering coefficient, which is the ratio between the bubble scattering cross-section (a_{s}) and the bubble geometrical cross-section area (a_{b}).

$$K_{\mathrm{a}} = \frac{a_{\mathrm{s}}}{a_{\mathrm{b}}} \tag{S12}$$

Bubble scattering is assumed to be wavelength-independent, which is validated by measuring the transmittance through our (photo)electrochemical cell with and without bubble formation at 1 bar

(see Fig. S9). Finally, the bubble-induced optical loss ($f_{\text{opt. loss}}$) is therefore given by the following:

$$f_{\text{opt. loss}} = 100\left(1 - f_{\text{opt. trans.}}\right)\ [\%] \tag{S13}$$

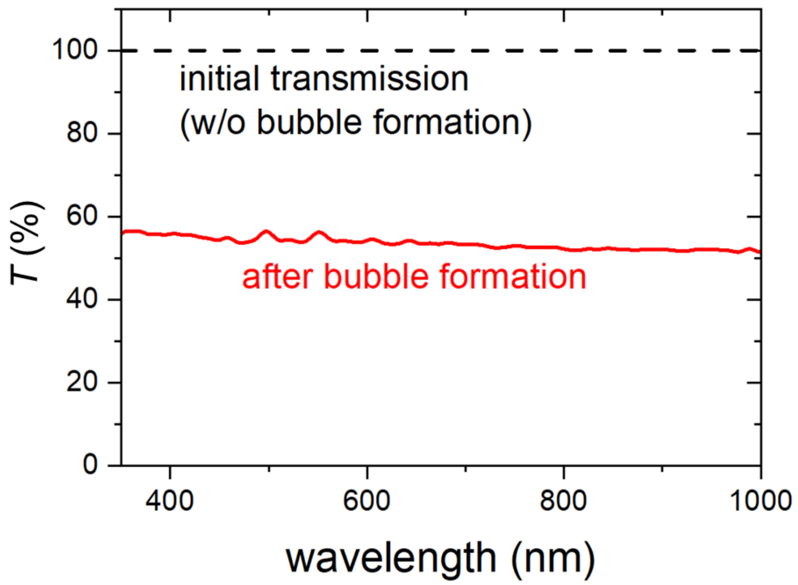

Figure S9. Transmittance measured through a transparent (photo)electrochemical cell at 1 bar without and with bubble formation. 20-pt FFT filter was applied to the spectrum with bubble formation to reduce the signal noise.

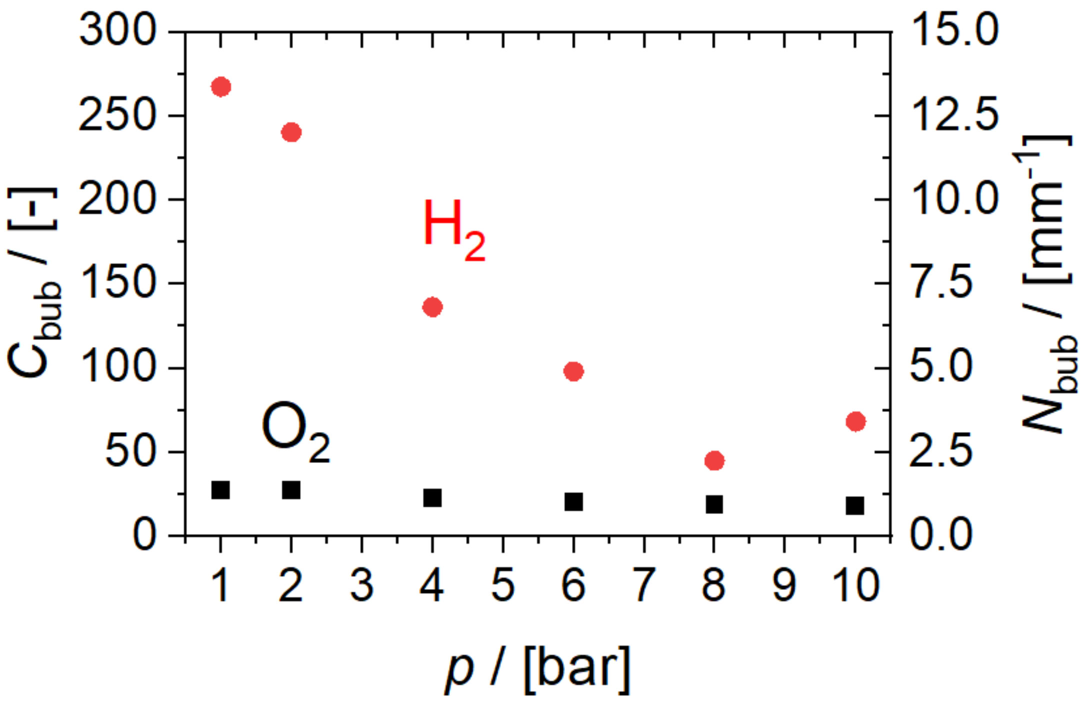

Figure S10. The calculated total number (C_{bub}) and number density (N_{bub}) of H_2 and O_2 bubbles as a function of the operating pressure. Note that the unit for N_{bub} is mm^{-1} instead of mm^{-2} here since our model is a 2D model, which means the number density of bubbles is considered along the electrode length (L_y).

Supplementary Note 4 – Estimation of the bubble coverage overpotential

Bubble coverage (Θ) on the surface of the (photo)electrode was calculated by taking the line integral of the gas volume fraction from our multiphysics simulation results (e.g., see Fig. 4). Results obtained from various operating pressures were used to generate the dependence of pressure on the bubble coverage, as shown in Fig. S11a. We only considered the case of device angle (θ) = 90° (i.e., vertical orientation), as it represents the worst-case scenario for bubble coverage. The bubble coverage induced overpotential, V_{Θ}, was then calculated based on the following equation:[8]

$$V_{\Theta} = -\frac{2.3RT}{\alpha F}\log_{10}(1-\Theta) \qquad \text{(S14)}$$

where the term $2.3RT/\alpha F$ is the Tafel slope of the (photo)electrocatalysts, which is determined by the reaction mechanism and the rate-determining step. Here, we take a Tafel slope value of 120 mV dec^{-1}, as reported for the HER on Pt and the OER on NiO_x under neutral pH conditions.[9,10] Fig. S11b shows the plot of the bubble coverage induced overpotential, V_{Θ}, as a function of pressure.

Note that these estimates assume that the contact angle of the bubble is independent of the change in hydrostatic pressure. The assumption is reasonable, as supported by a recent experimental study of a water-N_2-PTFE system.[11] In that study, the contact angle of the water droplet was measured under various pressures and temperatures, and pressure elevation up to 50 bar only introduced minor changes to the contact angle.

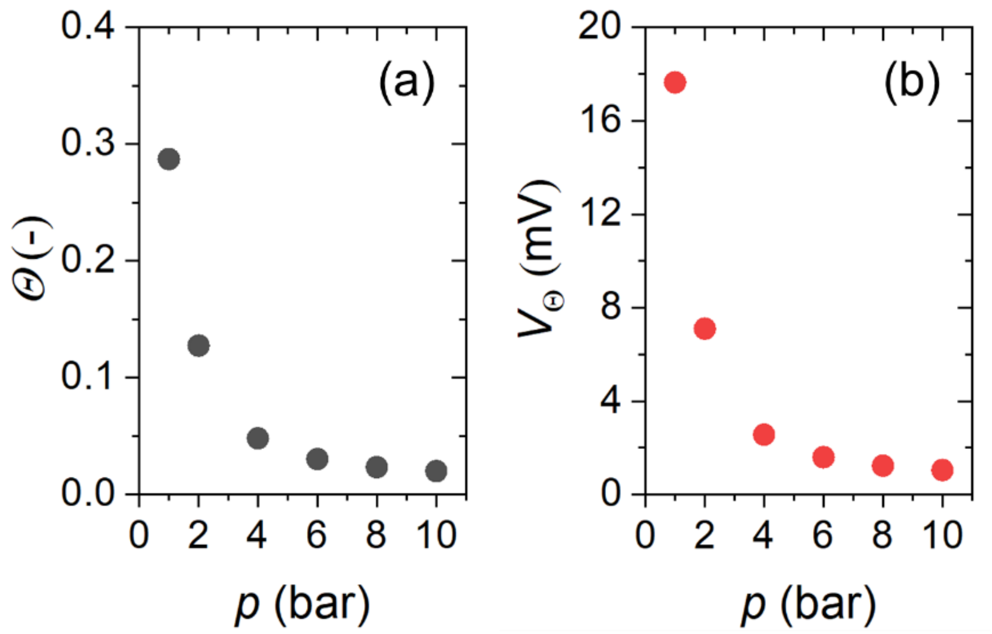

Figure S11. (a) Bubble coverage (Θ) of electrode surface as a function of pressure as obtained from our multiphysics simulations (Fig. 3a-e) at device angle (θ) 90°. (b) The resulting bubble overpotential (V_Θ) calculated from the data of Θ in (a) and the empirical relationship reported by Vogt.[8]

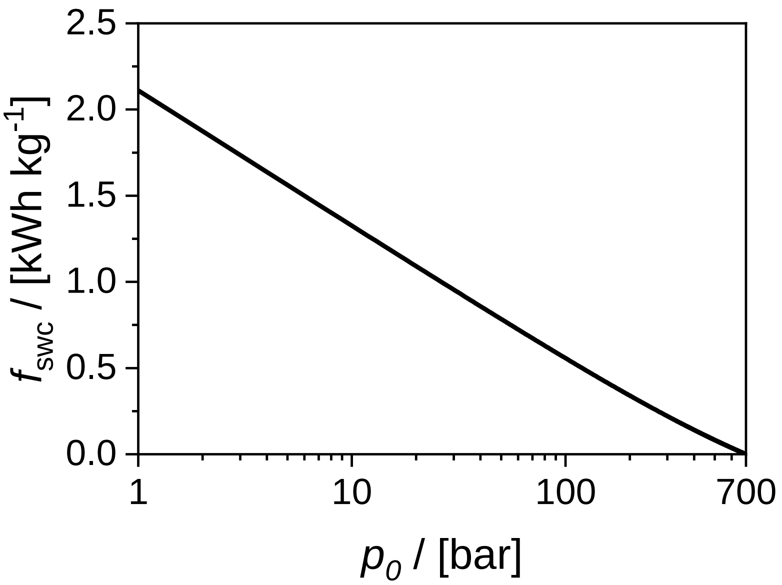

Figure S12. Specific work needed (f_{swc}) for compressing 1 kg hydrogen from p_0 (collection pressure from the PEC water splitting device) to 700 bar.

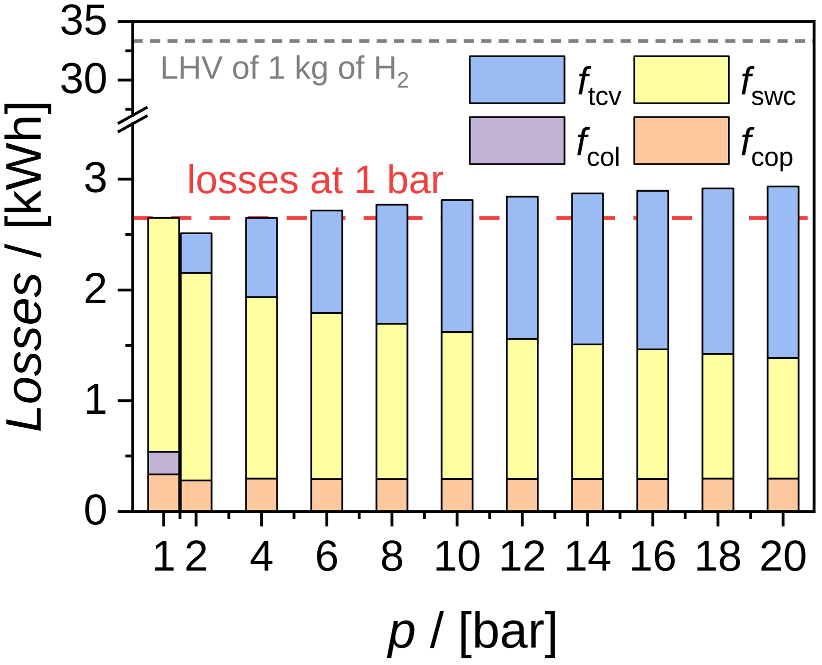

Figure S13. Energy losses associated with operating PEC water splitting cells at various pressures，in absence of the bubble scattering induced optical loss.

Supplementary Note 5 – Influence of pressure on the diffusion coefficient

We investigated whether the variation of operating pressure would have any impact on the values of diffusion coefficient considered in the electrolyte. Diffusion coefficient is related to a number of parameters according to the Stokes-Einstein relationship: [12,13]

$$D_{\mathrm{m}} = \frac{k_{\mathrm{B}}T}{3\pi d\mu_{\mathrm{L}}} \quad \text{(S15)}$$

where k_{B} is the Boltzmann constant, d is the Stokes diameter or hydrodynamic diameter, and μ_{L} is the dynamic viscosity of the electrolyte. Considering that diluted solutions are used in our case, the pressure-dependence of μ_{L} of our electrolyte should be similar to that of water. We therefore plotted the μ_{L} of water as a function of temperature for different pressure, as shown in Figure S14. The curves at different pressure (up to 10 bar, i.e., our pressure range of interest) practically fall on top of each other, suggesting that the dynamic viscosity of water can be considered to be pressure-independent in our case. Since the other parameters in equation S15 are also pressure-independent, we can consider the diffusion coefficients involved in our simulations to be unaffected with the operating pressure. The diffusion coefficient values listed in Table S2 were therefore used at all pressure.

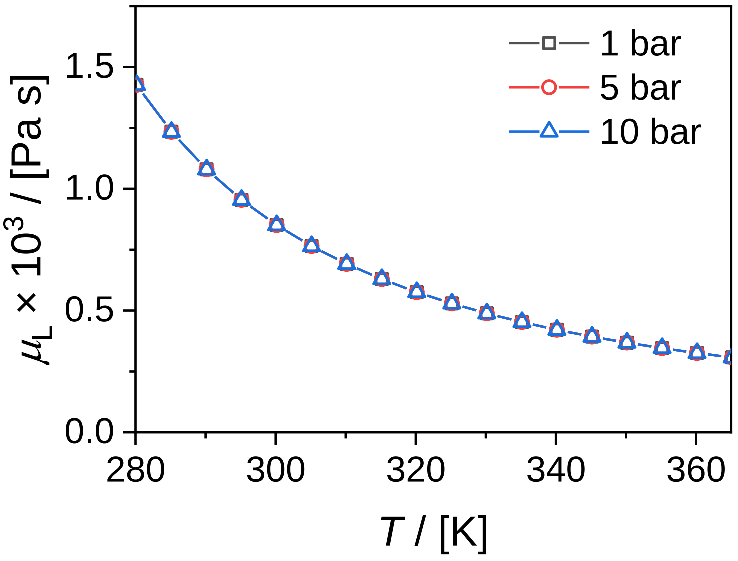

Figure S14. Dynamic viscosity of water (μ_{L}) as a function of temperature at 1, 5, and 10 bar. The curves overlap with each other, indicating that μ_{L} is pressure-independent.

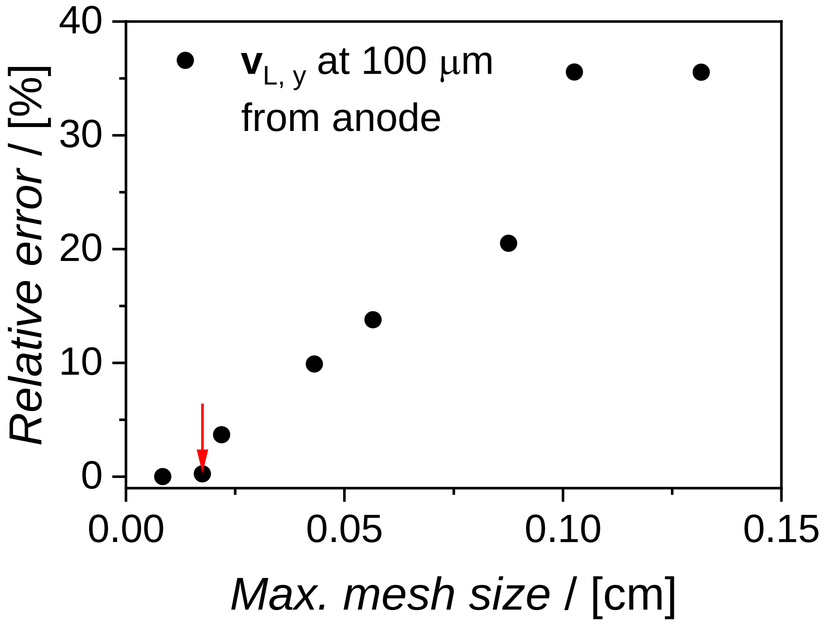

Figure S15. Relative error of the liquid velocity as a function of the maximum mesh size. The liquid velocity is considered in the *y*-direction ($\mathbf{v}_{L,y}$) at 100 μm from the anode. The red arrow indicates the mesh size chosen for our simulations.

Table S2. Variables considered in our model.

Variables	Value	Comments
p / [bar]	1, 2, 4, …, 10	p_out = 0, 1, 3, …, 9
L_x / [cm]	0.5	gap between the electrodes
L_y / [cm]	2	height of the electrodes
θ: tilt angle / [°]	90, 80, 70, …, 0	90° represents the vertical position
inlet velocity of the electrolyte / [cm/s]	3	

Table S3. Parameters used in our multiphysics simulations.

Parameters	Value	Info.	Ref.
R / [J/mol·K]	8.314	Gas constant	
$\mathbf{g}$ / [kg/s^2]	9.81	Gravitational acceleration	
F / [C/mol]	96485.3	Faraday constant	
T / [K]	298.15	Temperature	
M_{H_2} / [g/mol]	2	Molar mass of H_2	
M_{O_2} / [g/mol]	32	Molar mass of O_2	
ρ_L / [kg/m^3]	997.5	Density of the fluid	[14]
μ_L / [Pa s]	8.9 x 10^{-4}	Dynamic viscosity of the fluid	[14]
ρ_G / [kg/m^3]	Pressure dependent	Density of the gas	[14]
μ_G / [Pa s]	Pressure dependent	Dynamic viscosity of the gas	[14]
D_{H_2} / [m^2/s]	5.0 x 10^{-9}	Diffusion coefficient of dissolved H_2	[15]
D_{O_2} / [m^2/s]	2.4 x 10^{-9}	Diffusion coefficient of dissolved O_2	[15]
D_{H^+} / [m^2/s]	9.3 x 10^{-9}	Diffusion coefficient of dissolved H^+	[15]
D_{K^+} / [m^2/s]	1.96 x 10^{-9}	Diffusion coefficient of dissolved K^+	[15]
$D_{H_2PO_4^-}$ / [m^2/s]	0.85 x 10^{-9}	Diffusion coefficient of dissolved $H_2PO_4^-$	[15]
$D_{H_2PO_4^{2-}}$ / [m^2/s]	0.69 x 10^{-9}	Diffusion coefficient of dissolved $H_2PO_4^{2-}$	[15]
$c_{H_2,bulk}$ / [mol/L]	0	Initial bulk concentration of dissolved H_2	
$c_{O_2,bulk}$ / [mol/L]	0	Initial bulk concentration of dissolved O_2	
$c_{H^+,bulk}$ / [mol/L]	1.0 x $10^{-7.21}$	Initial bulk concentration of H^+	
$c_{K^+,bulk}$ / [mol/L]	1	Initial bulk concentration of K^+	
$c_{H_2PO_4^-,bulk}$ / [mol/L]	1	Initial bulk concentration of $H_2PO_4^-$	
$c_{H_2PO_4^{2-},bulk}$ / [mol/L]	1	Initial bulk concentration of $H_2PO_4^{2-}$	
K_{a2} / [mol/L]	1.0 x $10^{-7.21}$	2nd buffer equilibrium constant	
LHV / [kWh/kg]	33.33	Lower heating value of hydrogen	[15]

Table S4. Electrochemistry kinetic parameters used in our simulations.

Parameters	Value	Info.	Ref.
$\mathbf{j}_{0,\mathrm{OER}}$ / [mA/cm^2]	1 x 10^{-5}	Exchange current density for OER	[16,17]
$\mathbf{j}_{0,\mathrm{HER}}$ / [mA/cm^2]	1	Exchange current density for HER	[16,17]
$\alpha_{\mathrm{a,HER}}$ / [-]	0.5	HER anodic transfer coefficient	
$\alpha_{\mathrm{c,HER}}$ / [-]	0.5	HER cathodic transfer coefficient	
$\alpha_{\mathrm{a,OER}}$ / [-]	1.9	OER anodic transfer coefficient	
$\alpha_{\mathrm{c,OER}}$ / [-]	0.1	OER cathodic transfer coefficient	

Supplementary References

1 Leenheer, A. J. & Atwater, H. A. Water-splitting photoelectrolysis reaction rate via microscopic imaging of evolved oxygen bubbles. *J. Electrochem. Soc.* **157**, B1290 (2010).

2 Sillen, C. W. *The effect of gas bubble evolution on the energy efficiency in water electrolysis*, Eindhoven University of Technology, Netherlands, (1983).

3 Holmes-Gentle, I., Bedoya-Lora, F., Alhersh, F. & Hellgardt, K. Optical losses at gas evolving photoelectrodes: implications for photoelectrochemical water splitting. *J. Phys. Chem. C* **123**, 17-28 (2018).

4 Shibata, S. Supersaturation of oxygen in acidic solution in the vicinity of an oxygen-evolving platinum anode. *Electrochim. Acta* **23**, 619-623 (1978).

5 Vogt, H. On the supersaturation of gas in the concentration boundary layer of gas evolving electrodes. *Electrochim. Acta* **25**, 527-531 (1980).

6 Takahashi, T. *et al.* Global sea–air CO_2 flux based on climatological surface ocean pCO_2, and seasonal biological and temperature effects. *Deep-Sea Res. II: Top. Stud. Oceanogr.* **49**, 1601-1622 (2002).

7 Smith, F. L. & Harvey, A. H. Avoid common pitfalls when using Henry's law. *Chem. Eng. Prog.* **103**, 33-39 (2007).

8 Balzer, R. & Vogt, H. Effect of electrolyte flow on the bubble coverage of vertical gas-evolving electrodes. *J. Electrochem. Soc.* **150**, E11 (2002).

9 Shinagawa, T., Ng, M. T. K. & Takanabe, K. Electrolyte Engineering towards Efficient Water Splitting at Mild pH. *ChemSusChem* **10**, 4155-4162 (2017).

10 Zheng, J., Yan, Y. & Xu, B. Correcting the hydrogen diffusion limitation in rotating disk electrode measurements of hydrogen evolution reaction kinetics. *J. Electrochem. Soc.* **162**, F1470 (2015).

11 Song, J.-W., Ma, M.-C. & Fan, L.-W. Understanding the temperature dependence of contact angles of water on a smooth hydrophobic surface under pressurized conditions: An experimental study. *Langmuir* **36**, 9586-9595 (2020).

12 Obata, K., Stegenburga, L. & Takanabe, K. Maximizing Hydrogen Evolution Performance on Pt in Buffered Solutions: Mass Transfer Constrains of H_2 and Buffer Ions. *J. Phys. Chem. C* **123**, 21554-21563 (2019).

13 Xing, W. *et al.* Oxygen solubility, diffusion coefficient, and solution viscosity. in *Rotating Electrode Methods and Oxygen Reduction Electrocatalysts* (eds Wei Xing, Geping Yin, & Jiujun Zhang) 1-31 (Elsevier, 2014).

14 Lemmon, E., Bell, I. H., Huber, M. & McLinden, M. NIST Standard Reference Database 23: Reference Fluid Thermodynamic and Transport Properties-REFPROP, Version 10.0, National Institute of Standards and Technology. *Standard Reference Data Program, Gaithersburg* (2018).

15 Haynes, W. M., Lide, D. R. & Bruno, T. J. *CRC Handbook of Chemistry and Physics*. (CRC Press, 2016).

16 Haussener, S. *et al.* Modeling, simulation, and design criteria for photoelectrochemical water-splitting systems. *Energy Environ. Sci.* **5**, 9922-9935 (2012).

17 Obata, K. & Abdi, F. F. Bubble-induced convection stabilizes the local pH during solar water splitting in neutral pH electrolytes. *Sustain. Energy Fuels* **5**, 3791-3801 (2021).
