# Supplementary material for: Assessing elevated pressure impact on photoelectrochemical water splitting via multiphysics modeling
Source: Nat Commun. 2024 Jun 10;15:4944. doi: 10.1038/s41467-024-49273-2 (PMC11164907; doi:10.1038/s41467-024-49273-2)
Supplement: Supplementary file 3 — Description of Additional Supplementary Files [file 41467_2024_49273_MOESM3_ESM.pdf]

Description of Additional Supplementary Files

Supplementary Movie 1: Side-view of O2 and H2 bubble curtains generated from Pt/FTO anode (A) and cathode (C), respectively. The experiment was performed in 1 M KOH electrolyte solution at 10 mA cm-2 and 1 bar.

Supplementary Movie 2: Side-view of O2 and H2 bubble curtains generated from Pt/FTO anode (A) and cathode (C), respectively. The experiment was performed in 1 M KOH electrolyte solution at 10 mA cm-2 and 2 bar.

Supplementary Movie 3: Side-view of O2 and H2 bubble curtains generated from Pt/FTO anode (A) and cathode (C), respectively. The experiment was performed in 1 M KOH electrolyte solution at 10 mA cm-2 and 3 bar.

Supplementary Movie 4: Side-view of O2 and H2 bubble curtains generated from Pt/FTO anode (A) and cathode (C), respectively. The experiment was performed in 1 M KOH electrolyte solution at 10 mA cm-2 and 4 bar
